# Heterodissemination: precision insecticide delivery to mosquito larval habitats by cohabiting vertebrates

**DOI:** 10.1038/s41598-021-93492-2

**Published:** 2021-07-08

**Authors:** Isik Unlu, Ary Faraji, Yi Wang, Ilia Rochlin, Randy Gaugler

**Affiliations:** 1 Center for Vector Biology, Rutgers University, 180 Jones Avenue, New Brunswick, NJ 08901 USA; 2 Salt Lake City Mosquito Abatement District, 2215 North 2200 West, Salt Lake City, UT 84116 USA; 3 Mosquito Control and Habitat Management Division, 8901 NW 58th Street, Miami, FL 33178 USA

**Keywords:** Biological techniques, Ecology

## Abstract

Conventional larvicide delivery strategies originally developed for permanent and floodwater mosquitoes have proved suboptimal in the small, scattered, and cryptic larval habitats preferred by container-inhabiting *Aedes* mosquitoes. New methods such as autodissemination, wherein adult mosquitoes spread insecticides to their own larval habitats, have been under study. Another novel delivery method termed heterodissemination, i.e. larvicide delivery by other species sharing the same habitats, has also been proposed. We conducted a proof-of-concept study with four independent experiments using American bullfrogs (*Lithobates catesbeianus*) and green frogs *Lithobates clamitans* as carriers of pyriproxyfen, an insect growth regulator, under semi-field conditions in three different locations, two in New Jersey, and one in Utah. Frogs with attached slow-release pyriproxyfen tablets were introduced into outdoor enclosures with water containers. Water samples from the containers were periodically tested using larval *Aedes albopictus* and *Culex pipiens* mosquitoes to assess mortality and percent eclosure inhibition. Overall pupal mortality [95% credible intervals] estimated by Bayesian analysis for the treatment group was 73.4% [71.3–75.2] compared to 4.1% [2.9–5.5] for the control group. Mortality within treatment groups in four different experiments ranged from 41 to 100%, whereas control mortalities ranged from 0.5% to 11%. We conclude that heterodissemination is a promising and effective approach deserving of further study.

## Introduction

Globalization and urbanization have facilitated the dispersal of container-inhabiting invasive mosquitoes from the genus *Aedes*, such as *Aedes aegypti* L. and *Aedes albopictus* (Skuse)^1^. These mosquitoes transmit many established and emerging arboviruses including Zika, yellow fever, dengue, and chikungunya^2,3^. Vector control remains the cornerstone of preventative and reactive measures to mitigate these diseases. However, conventional mosquito control depends heavily on source reduction and area-wide insecticide applications, methods that were largely developed to manage mosquitoes in permanent and floodwater habitats^4–6^. These habitats are typically extensive, easily identifiable, and accessible^7^. They contrast sharply with larval habitats that invasive *Aedes* species use i.e., small, artificial containers that are widely scattered in residential areas with limited access to vector control personnel^3,8^. As a result, the best container-inhabiting mosquito management approach, source reduction, is not a realistic option for control of invasive *Aedes* populations^9,10^. Targeted application of larvicides and pupacides by backpack sprayers can be effective on a small scale, but they are labor intensive and require access to private residential properties^5,6^. The most common area-wide larvicide strategy employs truck-mounted applications^11,12^. However, the affinity of invasive *Aedes* mosquitoes for cryptic larval habitats shielded from insecticide droplet penetration by foliage or man-made structures can limit the impact of these control measures on immature mosquito populations^13,14^. One example of a cryptic larval habitat is corrugated extension downspouts, which provide only a single small opening for spray droplets to enter^15^. Such containers, potentially harboring high numbers of *Ae*. *albopictus* mosquitoes, can be ubiquitous in suburban environments, but inaccessible to conventional control techniques.

The unique management challenges posed by invasive *Aedes* mosquitoes suggest that conventional strategies and tactics developed for permanent and floodwater mosquitoes are suboptimal for use in small, scattered, cryptic habitats^14^. Itoh^16^ recognized the need for novel approaches to manage these habitats when he proposed that adult *Ae. aegypti* females could be harnessed as a vehicle to contaminate larval habitats with insecticides. This method, designated “autodissemination”, exploits gravid mosquito oviposition behavior by attraction to stations that contaminate the female with a larvicide, which is subsequently transferred to larval habitats^17,18^. In sharp contrast to area-wide larvicide sprays, autodissemination permits precision mosquito control with the mosquitoes themselves finding and treating cryptic habitats, with comparatively minute amounts of insecticides. Small and large-scale field tests of autodissemination have supported the concept that needs further field assessment under more realistic conditions^19–22^. Modifications of autodissemination methods, for example by contaminating and releasing laboratory-reared gravid mosquitoes^23^, or broadcast applications of insecticides to contaminate mosquito resting sites^24^, have also shown promise for mosquito control.

The autodissemination concept has recently been further extended to “heterodissemination”, defined as the transfer of insecticides into larval habitats using cohabitants but not conspecifics as the delivery vehicles^25^. Invasive *Aedes* share their container larval habitat with other species, particularly chironomid (non-biting) midges in the Dipteran family Chironomidae. The container-inhabiting midge *Chironomus decorus* Johannsen was reared in the laboratory and females coated with the juvenile hormone mimic pyriproxyfen^25^. Contaminated midges released under semi-natural conditions and in a small field study successfully delivered pyriproxyfen into immature mosquito habitats, causing 70–90% pupal mortality^25^.

Multiple invertebrate and vertebrate species share habitats with immature mosquitoes. Ornamental plantings of bromeliads, for example harbor a rich fauna of insects and amphibians, including frogs, as well as arguably the most important mosquito disease vector, *Ae*. *aegypti*^26^. Frogs might be exploited to deliver insecticides, such as an insect growth regulator (IGR), into highly cryptic habitats^27,28^. The current study is a proof of concept investigation for our hypothesis the possibility of using a non-insect cohabitant for a pyriproxyfen heterodissemination approach. We attempted to extend the heterodissemination tactic by assessing whether frogs with attached pyriproxyfen-impregnated tablets could transport lethal IGR concentrations to shared mosquito habitats. This is the first report of using a non-insect cohabitant for a heterodissemination approach to deliver an insecticide into mosquito aquatic habitats.

## Materials and methods

We conducted four field cage experiments in New Jersey (two locations) and Utah (one location). The study areas and the cages differed among the experiments, but the same protocol was followed for collecting water samples to evaluate efficacy of the heterodissemination approach. For all locations, we followed guidelines in the Guide for the Care and Use of Laboratory Animals^29^ as approved by the Animal Use Committee of Rutgers University under protocol No. 86–129. The experimental setup is summarized in Table 1.

**Table 1:** Summary of experimental design conducted in New Jersey and Utah, in 2017.

Experiment/ setup	NJ1	NJ2	NJ3	UT
Space, m^2^	300	8	8	300
Frog spp	*Rana clamitans*	*Rana clamitans*	*Rana clamitans*	*Lithobates catesbeianus*
# frogs released	10	1	1	5
Date of frog release	30-Jun	1-Jul	3-Sep	14-Jul
Date sampled	10 Jul, 24 Jul, 11 Aug, 4 Sep	7 Jul, 14 Jul, 28 Jul	10 Sep, 17 Sep, 1 Oct	21 Jul, 4 Aug, 18 Aug, 1 Sep
Mosquito spp	*Ae*. *albopictus*	*Ae*. *albopictus*	*Ae*. *albopictus*	*Cx*. *pipiens*
# larvae per replica	20	20	20	11–30 (variable)
# replicas treatment	10	6	6	10
# replicas control	3	3	3	3

### Field Cage Trial, Trenton, New Jersey (experiment NJ1)

**Figure 1:**
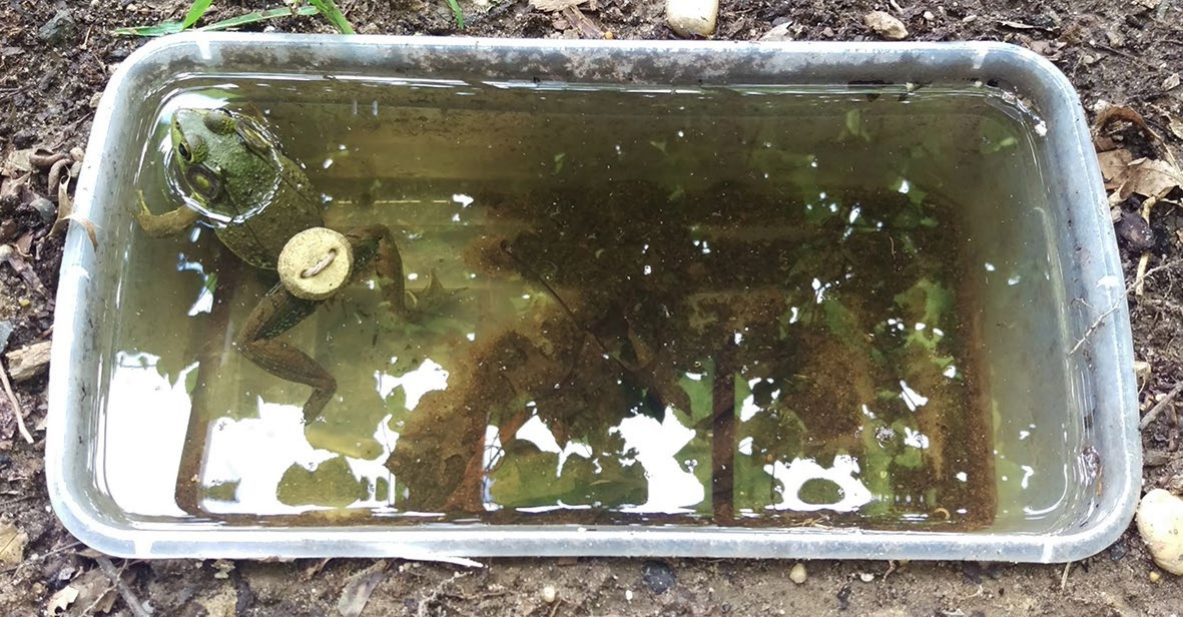
A slow release tablet formulation attached to the lower abdomen of a green frog, *Lithobates clamitans,* collected from a pond at Trenton, NJ before release into the enclosure (NJ experiments).

A large tunnel field cage (50 m × 3 m × 2 m or 300 m^3^) constructed of nylon mesh was placed in a grassy field at Mercer County Mosquito Control, Ewing, New Jersey, USA . The tunnel was placed as a straight line and had partial shade at each terminus. Ten plastic containers (32 × 18 × 11 cm) were buried at ground level and distributed equidistant at 9 m intervals. Each container was filled with six liters of dechlorinated water and replenished as needed. Ten green frogs, *Lithobates clamitans* (Latreille), were collected from a pond in Trenton, New Jersey and a pyriproxyfen tablet was attached to the lower abdomen using a cotton string before being released into the enclosure (Fig. 1). Each week, 10 crickets were collected using a sweep net and released in the enclosure to provide food for the frogs. Water samples (250 ml) were collected four times (Table 1) and used in bioassays (10 cups).

### Field Cage Trial, New Brunswick, New Jersey (experiments NJ2 and NJ3)

An enclosure was created by burying six (0.06 × 1.2 × 2.4 m) pressure treated timbers into the ground to serve as a frame. Nylon window screen sealed the enclosure bottom (4 × 2 x 1 m). Six plastic containers (32 × 18 x 11 cm) were buried at ground level and equidistant and 0.4 m from each other. Each container was filled with dechlorinated water. One green frog, *Lithobates clamitans*, was collected from a pond in Trenton, NJ and a slow release tablet was attached to the lower abdomen using a string before being released into the enclosure (Fig. 1). From each container, 250 ml of water was sampled as shown in Table 1 and used in bioassays (6 cups in each experiment).

### Field Cage Trial, Salt Lake City, Utah (experiment UT)

**Figure 2:**
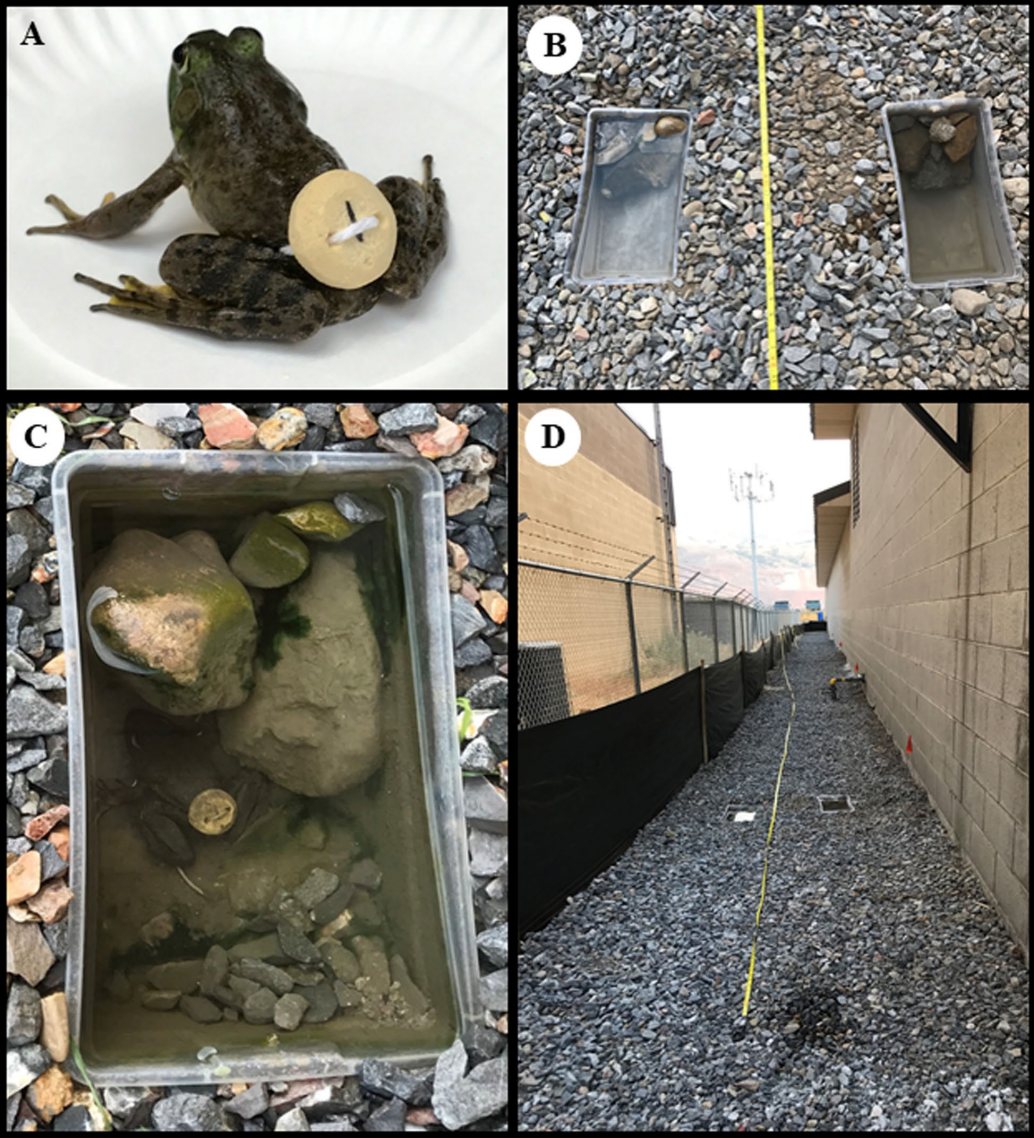
Field-cage experimental design for heterodissemination studies of pyriproxyfen using American bullfrogs (*Lithobates catesbeianus*) in Utah: (**A**) Frog with an attached pyriproxyfen tablet; (**B**) Plastic containers placed within the enclosure; (**C**) Frog resting within field-cage containers; (**D**) Semi-field experimental setup.

A field cage experiment was also conducted on the shaded north side of an industrial building in Salt Lake City, Utah. A 300 m^2^ (6 × 50 m) test enclosure was created with a one m high black silt fence (Home Depot, Atlanta, GA, USA) to retain the frogs . Ten plastic containers roughly 32 × 18 × 11 cm containing six liters of water were placed as pairs in the ground at 3, 9, 15, 27, and 37 m from the enclosure entry (Fig. 2). Rocks and pebbles were rinsed and placed in each container to facilitate frog entry and exit. American bullfrogs, *Lithobates catesbeianus* (Shaw), were purchased from Carolina Biological Supply (Burlington, NC) and a slow release tablet was attached to the lower abdomen with a cotton string before release into the enclosure. Ten house crickets, *Gryllodes sigillatus* (Walker) purchased from a local pet store were released weekly into the enclosure to provide food for the frogs. Five frogs were released into the enclosure one week prior to initial sampling and the subsequent samples collected four times (Table 1) for use in bioassays (10 cups).

### Slow-release insecticide tablet

A slow-release solid tablet formulation of pyriproxyfen was developed using a silicone mold. The mold plate (diameter 0.45 × 2.7 cm; weight 1.81 ± 0.08 g) was filled with 58.8% polyurethane casting resin, 20% methylated seed oil; 12.9% pyriproxyfen, and 8.3% polysorbate 20. Two holes (3 mm diameter) were formed in the tablet center with a distance of 1.4 cm to each other. The holes were used to attach the formulation to the dorsal posterior frog abdomen with string.

### Bioassays

Determination of pyriproxyfen activity via bioassays was conducted as previously described (Wang et al., 2014). Briefly, the 250 ml water samples were transported to the laboratory and the water was filtered into a cup through a paper towel to remove debris or other contaminants. Three cups containing 250 ml of dechlorinated water kept outside the experimental closures were used as controls for each experiment. Twenty *Ae. albopictus* (Mercer County, New Jersey) or 11–30 *Cx*. *pipiens* (Salt Lake City, Utah) third instar larvae were added to each cup, covers were affixed after cutting three slots (3–4 cm), and the cups were incubated at 26 ºC and 16:8 L:D photoperiod. Yeast (30 mg/L) was provided at 2-day intervals, water was replenished, dead larvae/pupae and emerged adults were removed and recorded until all individuals had emerged or died. Incomplete emergence of adults with attached exuvia was recorded as dead pupae.

### Mosquito colonies

All mosquito larvae used for New Jersey experiments (NJ1, NJ2, NJ3) were third instar *Ae. albopictus* obtained from a colony at the Center for Vector Biology at Rutgers University^22^. The colony was maintained at 26 ± 1 °C, 75% RH, and 16:8 h L:D. Restrained guinea pigs were used to provide a blood meal for the female mosquitoes (Rutgers University Animal Use Protocol #86–129). Eggs were collected inside the colony cages on oviposition papers and hatched as needed. Larvae were reared in enamel trays (ca. 200 larvae/tray) in 1 L of deionized water with 0.3 g of Brewer’s yeast provided on alternate days^30^. For the Utah study (UT), *Cx. pipiens* egg rafts were collected from the field at the Salt Lake City Mosquito Abatement District. Egg rafts were placed in individual pans for hatching, identified to species, and reared in enamel trays (ca. 200 larvae/tray) in 1 L of dechlorinated tap water with 0.3 g of Brewer’s yeast provided as food on alternate days. The colony was maintained at 26 ± 1 °C, 75% RH, and 16:8 h L:D. Only third instars were used in the bioassay experiments^31^.

### Statistical analysis

All statistical analyses used R v. 3.6.1^32^. Pupal mortality data was analyzed by generalized linear mixed effects model in package lme4 v. 1.1-21^33^. The *P*-values were obtained by likelihood ratio tests comparing the full model with and without the effect in question^34^. Post hoc tests were performed by Tukey test using the package “lsmeans” v. 2.30-0 comparing least square means adjusted for means of other factors in the model^35^. To check the model’s assumptions, residual plots were visually inspected for obvious deviations from homoscedasticity or normality.

The full mixed effects model included the interaction term of treatment and experiment as the fixed effects to account for potential differences among four independent experiments.

$$ {\text{Mortality }}\sim {\text{ Treatment }}*{\text{ Experiment }} + {\text{ }}\left( {{\text{session}}|{\text{container}}} \right) $$

Time point samples (i.e. sessions) nested within individual container represented repeated random effects to account for potential autocorrelation and the differences in response among different containers. The proportion of the dead or partially eclosed pupae per total was used as response variable in the model with a binomial distribution.

Mean pupal mortalities with associated 95% credible intervals for all experimental groups were estimated using the Bayesian approach because of a sample size and ease of interpretation. All models were fitted in JAGS v.4.3.0^36^ through package jagsUI v1.5.1^37^ using uninformative normally distributed priors for all coefficients including intercepts, sampling 10,000 posterior values from three Markov chains after a burn-in of 2,000 iterations. Convergence was inferred by R-hat values < 1.1.

## Results

**Figure 3:**
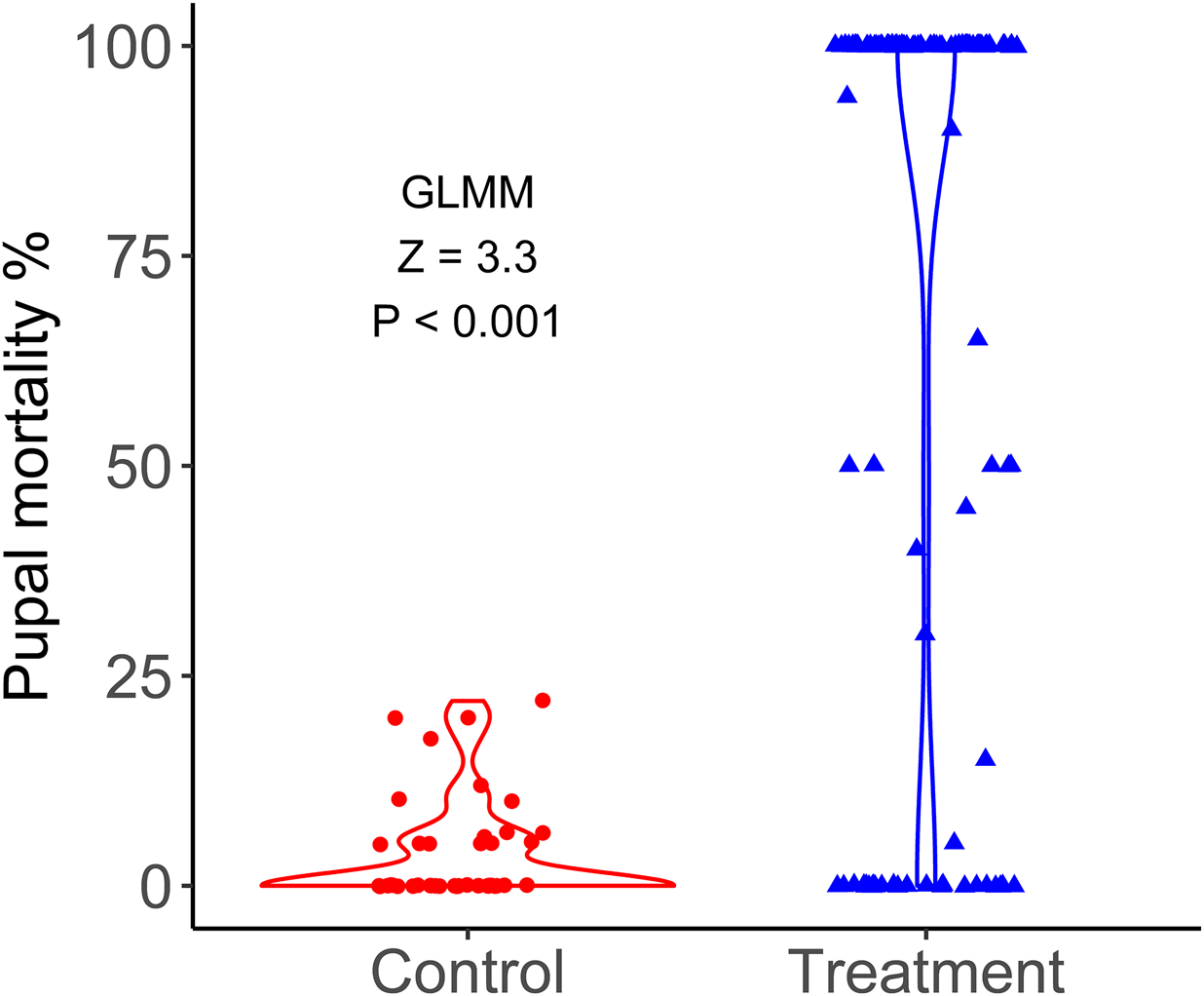
Comparison of pupal mortalities between the treatment and control groups. Violin plots show the full distribution of pupal mortality values in treatment and control groups. Dots indicate mortality measured in individual container at each time point. The overall treatment effect was statistically significant at Z = 6.1, *P* < 0.001 by generalized linear mixed effects (GLMM) model. The data from all four independent experiments were combined.

**Figure 4:**
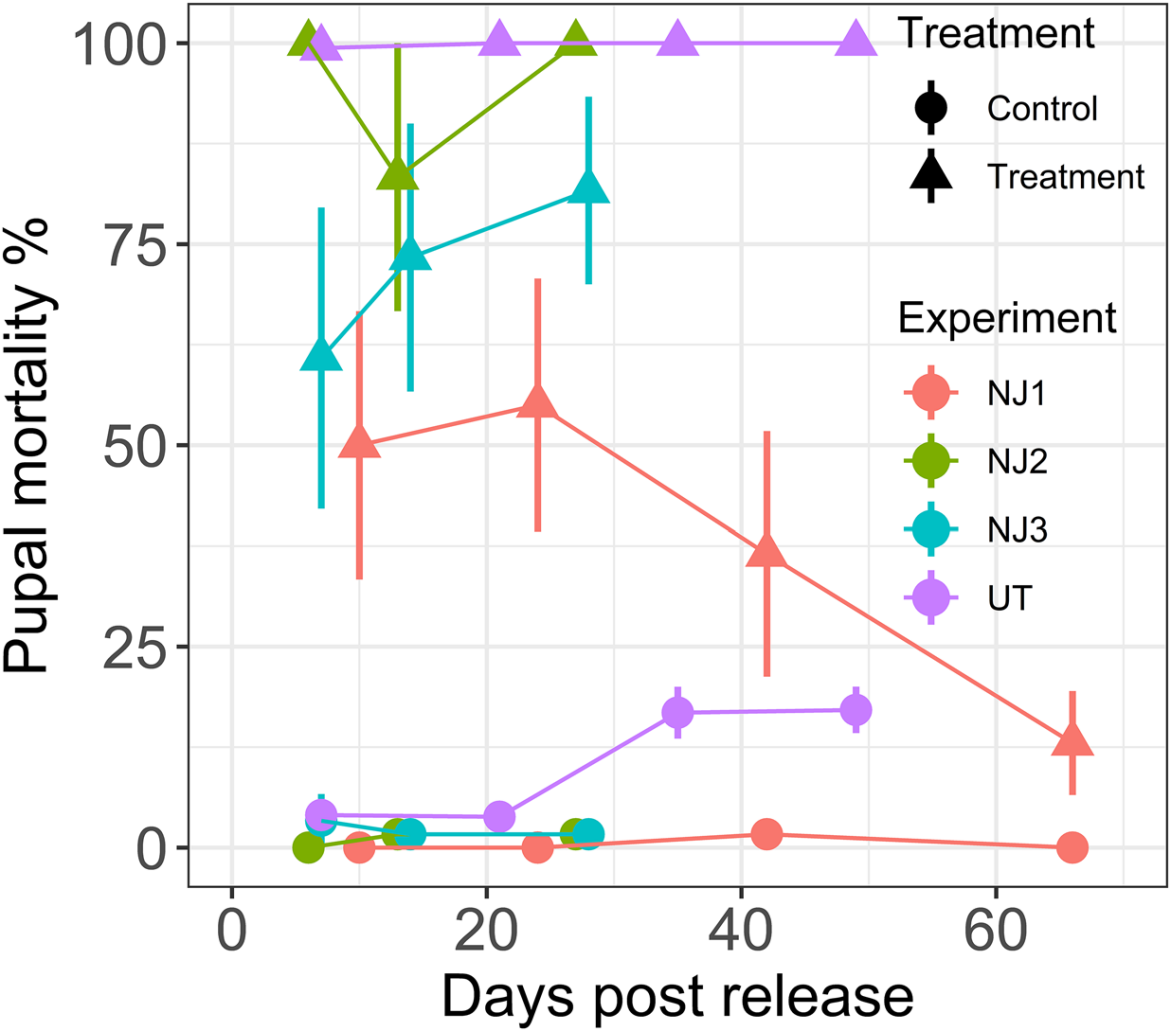
Average pupal mortality ± SE in each experiment over time. Four separate experiments were conducted, three in New Jersey (NJ1, NJ2, NJ3) and one in Utah (UT). *Aedes albopictus* larvae were used for New Jersey experiments, whereas *Culex pipiens* larvae were used in Utah. Line colors correspond to four separate experiments. Point symbols indicate treatment (triangles) or control (circles) group and days from the start of the experiments when the frogs were released (i.e. day 0).

A Bayesian t-test was used to estimate the posterior means and 95% credible intervals for the control versus the treatment groups combining the data from all four experiments (Figs. 3, 4). The overall pupal mortality [95% credible intervals] for the treatment group was 73.4% [71.3–75.2] compared to 4.1% [2.9–5.5] for the control group. The credible interval of the difference between the two groups, 69.3% [67.1–69.4] did not include zero. A Bayesian two-factor ANOVA was used to estimate the posterior means and 95% credible intervals for control and treatment groups in each of the four experiments (Table 2). Treatment group mosquito mortalities ranged from 41 to 100%., whereas control mortalities ranged from 0.5 to 11%.

**Table 2:** Pupal mortality posterior means and 95% credible intervals. Four separate and independent experiments were conducted, three in New Jersey (NJ1, NJ2, NJ3) and one in Utah (UT). *Aedes albopictus* larvae were used for New Jersey experiments, whereas *Culex pipiens* larvae were used in Utah. Mean mortality values fall within their corresponding credible intervals with a 95% probability.

Treatment	Experiment	Posterior mean	95% credible intervals	
Control	NJ1	0.4	0	1.5
Treatment	NJ1	40.6	37.6	43.8
Control	NJ2	1.0	0.1	2.9
Treatment	NJ2	94.8	92.3	96.9
Control	NJ3	2.2	0.6	4.7
Treatment	NJ3	72.2	67.4	76.7
Control	UT	11.1	7.6	15.3
Treatment	UT	99.7	99.2	100

Generalized linear mixed effects model (GLMM) was used to determine the treatment effect taking into consideration multiple experiments and time periods when the mortality measurements were recorded (i.e. sessions). Treatment and experiment interaction term was significant, *X*^2^ = 18.8, df = 3, *P* = 0.0003. Multiple comparisons among different experiments indicated that all three New Jersey experiments exhibited similar mosquito mortalities (all *P* > 0.05). However, the treatment mortality in Utah experiment was significantly greater than that in New Jersey for all comparisons (all *P* < 0.001). The main treatment effect was significant in the final model *Z* = 3.3, *P* = 0.00092 (Fig. 3). Mosquito mortalities measured in individual containers in the control group were low as indicated by a triangular shape of the violin plot with a wide base at 0%. For the treatment group, mosquito mortalities were mostly dichotomous, i.e. 100% or 0% with few intermediate values, resulting in an hourglass shape (Fig. 3). Most (70%) containers in the treatment group experienced high mortalities (> 90%) whereas over 80% of control containers experienced mortalities of less than 10%.

When pupal mortality was measured over time (Fig. 4), no discernable reduction was observed in three out of four experiments (NJ2, NJ3, UT) over periods of time ranging from 28 to 49 days in New Jersey and Utah, respectively. The downward trend was only present in the longest duration experiment (NJ1, Fig. 4), where the average treatment mortality declined from the peak of 55% at day 24, to 36.5% at day 42, and eventually to 13% at day 66 (Fig. 4). Control group mortalities were generally below 5% with the exception of the Utah control group which experienced elevated mortalities on two occasions of approximately 17% assayed on day 35 and day 49 of the experiment.

## Discussion

Our proof of concept study clearly demonstrated efficacious control against immature mosquitoes using frogs as carriers to deliver an insect growth regulator insecticide to container habitats. When laboratory reared pyriproxyfen treated midges were released under laboratory or semi-field conditions, approximately 75%-90% of *Ae. albopictus* pupal mortality was observed^25^. Under natural conditions, releasing 400 IGR-carrying midges into a residential backyard resulted in approximately 75% pupal mortality. These control levels are nearly identical to those obtained in the present study which yielded 73.4% mortality^25^. Thus, a slow-release IGR formulation tablet attached to frogs delivered sufficient concentrations of the insecticide to the containers.

There was considerable variability among the experiments in space and time. These differences can be attributed to several factors. Pyriproxyfen was reported to cause significantly high emergence inhibition against *Culex* and *Aedes spp* compared to control groups, therefore, different frog species may have affected the observed efficacy^38^. Bullfrogs used for the Utah experiment are known as very active but also the most aquatic species spending more time in the water compared to the green frogs used in New Jersey^39^. It is possible that more active movement among the containers and spending more time in the water led to the observed highest control efficacy in Utah experiment. The pupal mortalities in the treatment group had a clear “hourglass” distribution suggesting ‘on’ (i.e. containers contaminated by visiting frogs) or ‘off’ (i.e. containers that had no contact with the IGR because they were not visited) mechanism of control. Pyriproxyfen efficacy also waned over time during the longest duration experiment, with noticeable reduction after approximately 6 weeks.

Pyriproxyfen is highly toxic to mosquito larvae, for example *Ae.* *albopictus* LC_50_ = 0.012 ppb^17^. However, this IGR is classified as a reduced risk insecticide that is non-toxic to birds or mammals^40^. Pyriproxyfen degrades quickly in water^41^ leading to the conclusion that pyriproxyfen was “highly compatible with non-target organisms present in mosquito breeding habitats”^42^. Pyriproxyfen is also exceptionally low risk for humans, with a recommended drinking water limit of 300 ppb, a much higher concentration than required for effective mosquito control^40^.

The effects of pyriproxyfen on frogs have not been sufficiently elucidated. Ose et al.^43^ reported that tadpoles of the African clawed frog, *Xenopus laevis*, exposed at 300 ppb for 22 days did not show excess mortality or abnormal behavior, and that the chemical was metabolized and excreted^43^. In contrast^44^, indicated that *Odontophrynus americanus* (Dumeril and Bibron) tadpole behavior was affected by chronic exposure^44^. To our knowledge, no published studies evaluated the impact of pyriproxyfen on adult frogs. Further evaluations of sublethal and chronic pyriproxyfen exposure on amphibians are warranted, but these toxicological assessments were beyond the scope of the present study. Future studies should be considered to examine the impact of pyriproxyfen on frog fecundity, longevity, and activity. Interestingly, there are introduced and invasive frog species that might serve as pyriproxyfen carriers under field conditions. Consider the Cuban tree frog, *Osteopilus septentrionalis* (Dumeril and Bibron), a highly invasive species in Florida and southeastern US^45^. These frogs have several desirable characteristics as potential heterodissemination carriers – ability to survive in urban areas, propensity to use containers, pools, and bird baths for development, and arboreal habits allowing this species to inhabit phytotelmata plants such as bromeliads^46^.Bromeliads are an important habitat for *Ae*. *aegypti* in Florida^28^. Treating bromeliads with conventional methods are inefficient due to the labor-intensive nature of this method^47^. Some products labeled for mosquito control, such as the oil-based Cocobear™ (Clarke Mosquito Control, Roselle, IL, USA), can be phytotoxic. With limited conventional control options, targeting bromeliads as a key habitat for immature *Ae*. *aegypti* in urbanized areas represents a great challenge. Delivery of IGRs using an invasive and ubiquitous frog species might provide an additional tool urgently needed for control of container-inhabiting *Aedes* species^28,48^.

Another potential target for heterodissemination are those mosquito species that thrive in permanent water habitats in disturbed areas such as *Anopheles* mosquitoes^49^. Although *Anopheles* spp. in Africa can inhabit large permanent water bodies which can be treated using area-wide methods^50^. The same species are found in high numbers in smaller more ephemeral larval habitats that are difficult to locate and treat effectively^40^. These temporary habitats, especially those in urbanized environments, may be good candidates for heterodissemination treatments. The heterodissemination approach evaluated in this study as a proof of concept deserves further investigation to assess efficacy under “real world” conditions. It remains to be determined whether it is can become a viable practice for mosquito control. Additional investments to develop novel techniques are essential for meeting the challenges of effective mosquito control around the world.
